# Understanding children’s perspectives of the influences on their dietary behaviours

**DOI:** 10.1017/S1368980022000404

**Published:** 2022-08

**Authors:** Mei Jun Chan, Gabrielle Wann Nii Tay, Gayatri Kembhavi, Jubilee Lim, Salome A Rebello, Hazyl Ng, Congren Lin, May C Wang, Falk Müller-Riemenschneider, Mary Foong-Fong Chong

**Affiliations:** 1 Saw Swee Hock School of Public Health, National University of Singapore and National University Health System, 12 Science Drive 2, 117549 Singapore, Singapore; 2 Centre for Evidence and Implementation, Singapore, Singapore; 3 Faculty of Science, National University of Singapore, Singapore, Singapore; 4 Health Promotion Board, Singapore, Singapore; 5 Department of Community Health Sciences, Fielding School of Public Health, University of California, Los Angeles, CA, USA; 6 Singapore Institute for Clinical Sciences, Agency for Science, Technology and Research, 30 Medical Drive, 117609 Singapore, Singapore

**Keywords:** Dietary behaviour, Children, Socio-ecological model, Qualitative, Focus group, Perceptions

## Abstract

**Objective:**

This study aimed to examine the intrapersonal, interpersonal, environmental and macrosystem influences on dietary behaviours among primary school children in Singapore.

**Design:**

A qualitative interpretive approach was used in this study. Focus group discussions guided by the socio-ecological model (sem), of which transcripts were analysed deductively using the sem and inductively using thematic analysis to identify themes at each sem level.

**Setting:**

Two co-educational public primary schools in Singapore.

**Participants:**

A total of 48 children (*n* 26 girls) took part in the semi-structured focus group discussions. Their mean age was 10·8 years (sd = 0·9, range 9–12 years), and the majority of the children were Chinese (*n* 36), along with some Indians (*n* 8) and Malays (*n* 4).

**Results:**

Children’s knowledge of healthy eating did not necessarily translate into healthy dietary practices and concern for health was a low priority. Instead, food and taste preferences were pivotal influences in their food choices. Parents had a large influence on children with regards to their accessibility to food, their attitudes and values towards food. Parental food restriction led to some children eating in secrecy. Peer influence was not frequently reported by children. Competitions in school incentivised children to consume fruits and vegetables, but reinforcements from teachers were inconsistent. The proximity of fast-food chains in the neighbourhood provided children easy access to less healthy foods. Health advertisements on social media rather than posters worked better in drawing children’s attention.

**Conclusions:**

Findings highlighted important factors that should be considered in future nutrition interventions targeting children.

Childhood is a time of rapid physical and psychosocial development and changes^(1)^. Optimal nutrition and the development of good dietary habits are crucial during this life stage as habits formed can track into adulthood^(2)^. Unhealthy dietary behaviours have shown to affect the growth and development of children and contributed to the increasing prevalence of childhood obesity and its associated morbidities such as diabetes, CVD and disordered eating^(3)^. Current literature reveals that many children globally are not meeting their recommended food and nutrient intakes^(4)^. Similar trends have been observed in Singapore, where diets of primary school children, aged 6–12 years old, were found to be lacking in fruits, vegetables and whole grains, and high in sodium and added sugar^(5)^. To curb the raising prevalence of childhood obesity in Singapore, which has increased from 11 % in 2013 to 13 % in 2017^(6)^, it is crucial to understand the factors influencing the dietary behaviours among children in Singapore. Such information can help inform future health promotion programmes on improving children’s dietary behaviours.

Extensive research has shown that children’s dietary behaviours are influenced by multiple factors^(7,8)^. Using socio-ecological frameworks, previous studies have examined multiple levels of influence and the interplay among these levels^(9)^. For example, on the intrapersonal level, children’s dietary choices were found to be intrinsically influenced by their knowledge, skills, taste preference and familiarity with the food^(9–11)^. Interpersonal influences often come from children’s families^(12,13)^ and peers^(14)^, while community influences include schools^(15)^ and the physical environment (e.g. the proximity of food stores)^(16)^, while macrosystem influences include policies, media and advertisements^(9)^. However, most of these studies have largely been based on quantitative studies and reports from parents^(7–9,13,14,16)^.

In recent years, qualitative studies conducted with children themselves have been gaining traction as responses from children have been found to be richer and more accurate than proxy reporting by parents^(17,18)^. Additionally, such methodology has shown to be particularly useful in revealing new knowledge about the diverse determinants of dietary behaviour and in elucidating culture-specific influences^(10,19)^. Research has also shown that most children aged 7 and above enjoyed providing their opinions and were able to provide accurate and useful information for informing interventions^(20)^. However, to date, studies examining perceptions of children have mostly been conducted in North America, Europe, UK and Australia^(7,12,18)^ and there is a paucity of such studies in the Asian context^(10,19)^.

A deeper understanding of the socio-ecological influences on dietary behaviours from children themselves can help inform the development of age- and culture-appropriate dietary interventions to support sustainable behavioural change. Using a socio-ecological framework, this qualitative study aims to explore primary school children’s perspective of the socio-ecological influences on their dietary behaviours in Singapore using focus group discussions (FGD).

## Methods

### Study design and participants

A qualitative interpretive approach was used in this study to allow for a richer understanding of the influences on children’s dietary behaviours^(21)^. Focus group discussions (FGD) were conducted with children as they have shown to be less intimidating to children than individual interviews and allows ideas and discussions that may not arise in individual interviews to be elicited^(20)^. This study is reported according to the Consolidated Criteria for Reporting Qualitative Research^(22)^.

All FGD took place between July and November 2018. Children aged 9–12 years were recruited using convenience sampling from 2 co-educational public primary schools in Singapore. This age range was chosen to better understand children’s views as this is when they start gaining more autonomy in their food decisions and a critical period for prevention and health promotion^(23)^. As most of the primary schools in Singapore are public schools, students from public schools were selected for this study so that findings are applicable to most children. Information sheet with parental consent and child assent form was provided to 530 students in one school and 220 students in another school. Those who were interested to participate (*n* 64) submitted their consent and assent forms to us through their teachers (See Supplementary Figure for participant flow). Children’s demographic data were also provided by parents when consent forms were submitted.

### Data collection

A semi-structured discussion guide, principally guided by the Social-Ecological Model (sem), was designed to facilitate the FGD. The discussion guide was reviewed by an experienced qualitative researcher (GK), and questions in the guide were refined after pilot-testing in a separate group of children (*n* 14) from each school a month before data collection commenced (see Supplementary Table for key questions asked during the FGD). Analysis of notes and memos was conducted in conjunction with data collection and thus enabled the questions in the guide to be refined iteratively^(24)^.

Eleven focus groups were formed. These children were grouped so that the focus groups were homogenous in age (9–10 years, 11 years and 12 years) and gender at each school (see Supplementary Figure for participant flow). Such arrangement was made to reduce the variation in cognitive, linguistic, social and psychological competencies among children in the groups^(20)^. Each focus group had a median of 5 children with numbers ranging from 2 to 7. When there were more than 7 interested students in a focus group, students were selected randomly to form the group. All FGD were conducted after school hours and within the schools, in rooms with minimal noise (e.g. a classroom or discussion room in the school library). A familiar adult (teacher) was present at the initial meeting with the children, but not involved in the FGD as the presence of an authority figure may influence children’s responses during the FGD. All FGDs were conducted by MJC, who received training and guidance on the conduct of FGDs with children from an experienced qualitative researcher (GK), and an assistant moderator (GWNT or JL) who helped with notetaking and time management. There were no pre-existing relationships between the researchers and the children.

Children were informed before data collection that there would be 2 sessions of FGD – one for the influences on physical activity, while another on dietary behaviours. Ten groups attended 2 sessions of FGD, and one group attended only one session where both physical activity and dietary behaviours were discussed, due to time limitations. Data saturation was reached by the end of data collection. This article will focus on the FGD findings pertaining to dietary behaviour as findings on physical activity are reported elsewhere^(25)^.

Before each FGD commenced, the background of the researchers and the reasons for the study were introduced to the children. Ground rules (e.g. being respectful to others’ opinions, that there are no right or wrong answers in the discussion) were also explained to the children. To sustain their interest and attention, activities such as sorting picture cards (e.g. with images of common foods consumed by children and images of common agents of socialisation like family, friends, teachers, etc) and scenario-based questions that were relevant to the topic were incorporated into each FGD^(20)^. Each FGD lasted between 45 and 100 min. Tokens of appreciation, in the form of stationery (e.g. markers and pens) and snacks (e.g. malted beverage, fruit juice, biscuits), were given to all children at the end of each session. All FGDs were recorded using Sony UX560F digital voice recorders.

### Data analysis

All audio recordings were transcribed verbatim without identifiers. Transcripts were then checked against their corresponding audio recordings and imported to NVivo (Version 12, QSR International) to organise the data. Two researchers (MJC and GWNT) conducted the analysis first deductively using the sem, and then inductively for themes in each sem level, following the thematic analysis guide by Braun and Clarke^(26)^. The researchers read the transcripts repeatedly and generated meaningful codes from the transcripts independently. These codes were then discussed between the researchers before classifying them into themes at each level of the sem. To ensure credibility, codes were deliberated between MJC, GWNT, JL and MFFC and consensus was reached after several iterations. Saturation was considered to have been achieved when no new themes were identified.

## Results

**Fig. 1:**
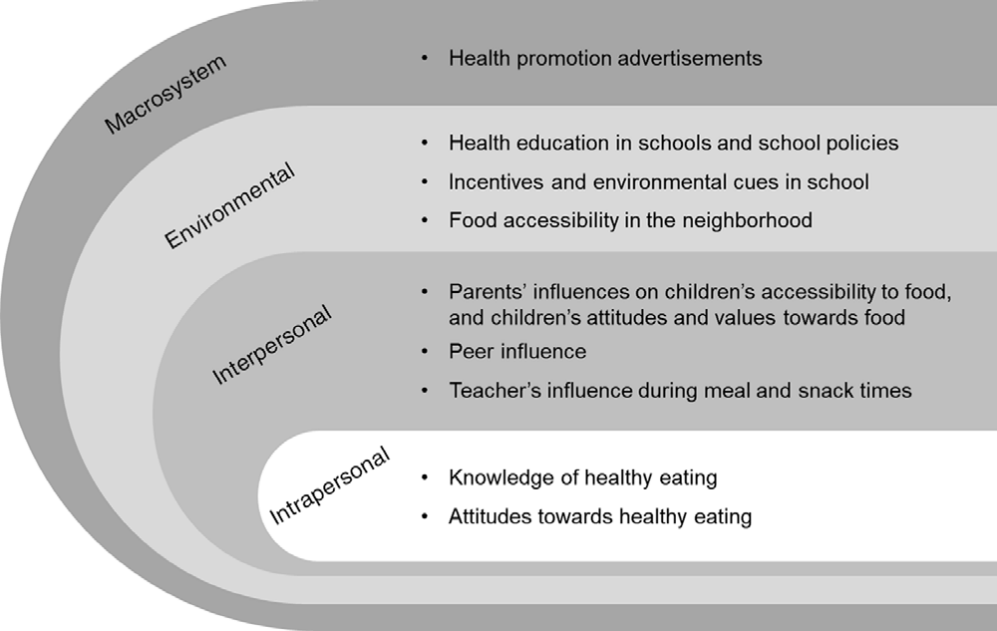
Key influences on children’s dietary behaviour summarised according to the socio-ecological model

Of those who consented to participate, 48 children (*n* 26 girls) took part in the semi-structured FGD. Their mean age was 10·8 years (sd = 0·9, range 9–12 years), and the majority of the children were Chinese (*n* 36), along with some Indians (*n* 8) and Malays (*n* 4). Sixteen children did not participate due to non-selection (*n* 10) or were not present during the FGD (*n* 6). Nine key themes relating to influences of children’s dietary behaviour emerged from the analysis and were classified according to the sem as shown in Fig. 1. Key quotes to illustrate each theme are presented in Table 1. This includes (1) knowledge and (2) attitudes towards healthy eating, (3) parents’, (4) peer and (5) teachers’ influences, (6) school’s education and policies, (7) incentives and environmental cues in school, (8) food accessibility in the neighbourhood and (9) health promotion advertisements. Generally, the themes that emerged were similar between boys and girls except for peer influence, which was more frequently discussed among the girls than boys. Themes were also similar across all age groups, but influence from the macrosystem level were not discussed among younger children (9–10 years old). It should also be noted that younger children tended to be more literal in their responses and provided less in-depth description and elaboration.

**Table 1:** Key themes and quotes from the focus group discussion data analysis on dietary behaviours

Theme	Representative quotes
Intrapersonal influences – knowledge of healthy eating	*Understanding of healthy eating* ‘(Vegetable is healthy) because it has vitamins and nutrients’ ‘(Fast food is not healthy) because it’s fried and it’s very oily… And it is very salty.’ (FG1, girls, 9–10 years old) ‘Healthy eating means eating fruits’, ‘Not eating any snacks’, ‘Vegetables’, ‘Drink water and do not drink sweet drinks’ (FG5, girls, 11 years old) *Recommended amounts of fruits and vegetables* ‘ (We need) at least half of the plate’, ‘or two portions’ (FG6, girls, 11 years old) ‘30 % rice, 20 % veggie, 50 % meat.’ (FG7, boy, 11 years old) *Importance of healthy eating* ‘You can grow very tall and smooth skin and pretty.’ (FG1, girl, 9–10 years old) ‘It (healthy foods) can help you grow taller, grow stronger.’ (FG3, boy, 9–10 years old) ‘Eating unhealthy foods everyday will grow very fat and die’ (FG7, boy, 11 years old) ‘I just think that like eating healthier will make me more healthy… because if you eat a lot of like unhealthy food, like a lot of salt, it will make your liver like maybe have uh cancer.’ (FG11, boy, 12 years old)
Intrapersonal influences – attitude towards healthy eating	*Consequences of unhealthy eating was not of immediate concern to them* Comparing Picture A (unhealthy foods) and Picture B (healthy foods) ‘B is healthy’ ‘Less sugar, less chance to get diabetes’ ‘But we still like A’ ‘because it’s yummy and taste better’. (FG2, girls, 9–10 years old) ‘Need to enjoy food. We only have less than 75 years to live. Eat all food we can; I don’t care (if) we get diabetes’ (FG10, boy, 12 years old) *Food preferences* ‘Most of the time, (if) the foods is not nice, it is healthy… Nice (foods) are not healthy.’ (FG8, boy, 11 years old) ‘If no chili in kangkong (a type of vegetable), I don’t want to eat.’, ‘Unless you can disguise until it looks like some other food then I will eat.’ (FG6, girls, 11 years old) ‘Sometimes (savory snacks and sweets are) too sweet, too salty or bitter. I don’t really like them anymore but sometimes if I want to eat, I try not to eat’ (FG1, girl, 9–10 years old) *Rationalizing unhealthier eating habits* ‘We can eat unhealthy food and then go exercise’ (FG10, boy, 12 years old) ‘Because there’s potato in fries. Potato is vegetable, vegetable is healthy.’ (FG6, girl, 11 years old) ‘Depends on mood. So, is it depending on whether you like the food or not, or whether you feel like eating or not? Or if I’m full, I just don’t care.’ (FG11, boy, 12 years old)
Interpersonal influences – parents’ influences on children’s accessibility to food and children’s attitudes and values towards food.	*Parental food restrictions* ‘I haven’t eaten at McDonalds for a long time’ ‘I only eat once a month’ ‘I only eat that on special days.’ (FG4, boys, 9–10 years old) ‘My father will scold me if I eat too much fast foods. He says, ‘You’ll grow fat.’’ (FG5, girl, 11 years old) ‘My mother won’t let me eat sweets and snacks, because too unhealthy. Fast foods are very, very, very, very rare.’ (FG8, boy, 11 years old). *Children’s response to food restrictions* ‘My mum doesn’t like us to eat snacks, but I listen to my mum so she’s happy with me’’ (FG1, girl, 9–10 years old) ‘My mum has this secret stash that she thinks no one knows but obviously everybody knows, so I go there every day for seaweed, nuts, and some chips’ (FG7, boy, 11 years old). ‘Secretly yeah, go to the *mama shop* (convenient store) to eat *Maggi mee* (instant noodles). Then tell my mum that my teacher uh, held me back late.’ (FG9, girl, 12 years old) *Parents provide healthy foods at home* ‘My father gets my family to eat fruits daily.’ (FG10, boy, 12 years old) ‘At home uh they will usually cook it with sauce… In school, they just serve plain vegetables.’ (FG6, girl, 11 years old) *Parents educate and prompt children to eat healthily* ‘Your arteries will be clogged and can die… (I learn) from my father’ (FG8, boy, 11 years old) ‘My mother says if you eat more sweets, (they) will grow more worms. These worms will eat your nutrients!’ (FG4, boy, 10 years old)) ‘My parents ask me to do this and that, like eat fruits and vegetables, but they themselves don’t do it’ (FG10, boy, 12 years old) *Children model after parents’ dietary habits* ‘My dad and I like to snack together.’ (FG5, girl, 11 years old) ‘(We) rarely eat fast food. ‘Cause we don’t - my parents don’t like eating oily food too’ (FG11, boy, 12 years old)
Interpersonal influences – peer influence	‘During snack time, my friend will, like, pass me a bit of sweets, and then I will (shows the action of sneaking sweet into mouth)’ (FG5, girl, 11 years old) (What do you usually eat with your friends when you go out?) ‘McDonalds’, ‘*Maggie mee* (instant noodles)’, ‘We’ll go 7-Eleven.’ (FG10, boys, 12 years old) ‘My friend will force me to eat’ (FG5, girl, 11 years old) ‘My friend try will try (the vegetable) first… (If) they say it’s nice, then I’ll eat’ (FG9, girl, 12 years old)) ‘Of course, every child eats fast foods!’ (FG1, girl, 9–10 years old)
Interpersonal influences – teacher’s influence during meal and snack times	‘Primary 1 to Primary 3 I ate *roti prata* (a type of fried bread) during recess. Then teacher says to change it, because *roti prata* contains a lot of oil. Then I changed to eating fish ball noodles’ (FG9, girl, 12 years old) ‘Oh, teacher always says, ‘Healthy things!’, but some still bring cake like those type of snacks. Then my teacher will take it away, like the chips they will take it away.’ ‘But my teachers won’t take, they will just scold you.’ (FG05, girls, 11 years old) ‘Miss A said uh no sweets, no potato chips, no unhealthy food, but we don’t care ‘cause not like the teacher is gonna do a spot check or something.’ ‘We ate (less unhealthy snacks) in front of our teacher before, but she didn’t say anything. So, I assume we can bring such snacks.’ (FG10, boys, 12 years old)
Environmental influences – Health education in schools and school policies	‘Yeah, in the Health Education and I remember last time in P5, they give us like this plate to follow, the plate is split into half and quarters.’ (FG11, boys, 12 years old) ‘We are forced to eat fruits every day… Every single plate of food, they will give you fruits.’ (FG6, girls, 11 years old) ‘Every time I get fruit, I throw away. The fruit is not nice.’, ‘I didn’t eat the vegetables because it’s hard to swallow. I just leave it there’, ‘I give to my friend’ (FG4, boys, 10 years old)
Environmental influences – incentives and environmental cues in school	‘You must pose while eating it, then Mr S would take photo. I think to show proof… then he will pass you the Fruity Veggie card. And then at the end (of the year), we see which class is the winner.’ (FG1, girl, 9–10 years old) ‘In our canteen, on the wall the school paste the banner of the My Healthy Plate.’ (FG9, girl, 12 years old) ‘They got all these posters all around the school but so sad, no one looks at them’ (FG7, boy, 11 years old)
Environmental influences – food accessibility in neighbourhoods	‘I just got to walk 5, 10 min to reach McDonald’s’ (FG1, girl, 9–10 years old) ‘I buy chips from either 7-Eleven or *mama shop* (convenient store). There is one, just across the street (from school).’ (FG6, girl, 11 years old) ‘Like after school, I go home, there’s a Mart on the way back and I always cross there when I go home, and I just go over there to get chips because it’s very convenient.’ (FG7, boy, 11 years old).
Macrosystem influences – health promotion advertisements	‘Advertisement at the bus stop for Let’s Beat Diabetes. They didn’t show the particular food, but they say the which food is healthy.’ (FG8, boy, 11 years old) ‘What less sugar, less salt, less oil ah, all these… Everywhere in Singapore will have one poster on healthy eating’ (FG9, girl, 12 years old)

### Intrapersonal influences

#### Knowledge of healthy eating

Most of the children demonstrated a good understanding of healthy eating and there was a consensus on the importance and benefits of healthy eating. When asked to define healthy eating, many children described foods that provide them with ‘nutrients and vitamins’, such as fruits, vegetables, carbohydrate and protein. Some mentioned limiting certain seasonings (e.g. sugar, salt and oil), as well as ‘junk’ and fast foods. When asked about the recommended amounts of fruits and vegetables one should consume, most of them were able to recall the correct portions stated on My Healthy Plate (a visual guide designed by the Singapore Health Promotion Board for creating balanced and healthy meals^(27)^). However, a few of them were unsure of the amounts or described the wrong portions. When asked about the importance of healthy eating, most children discussed how healthy eating helps reduce the risk of diabetes, cancer and growing ‘very fat and die’. A few of the children also alluded healthy eating to ‘grow taller and stronger’ and aesthetic reasons such as ‘smooth skin and pretty’.

#### Attitudes towards healthy eating

There were, however, inconsistencies between the children’s understanding of healthy eating and their corresponding attitudes. Only a few children reported that they adhered or tried to adhere to a healthy diet, while the rest of them felt that the consequences of unhealthy eating were not of immediate concern to them. Some also displayed an indifferent attitude, stating that their food choices should not be restricted, and they should be allowed the freedom to choose any food.

When it comes to food preferences, children generally value the taste of the food over their nutritional value. In most of the FGD, the children reported a preference for less healthy foods such as fast foods as they perceived these foods to be tastier than healthier foods such as vegetables, which many described as ‘tasteless’ and ‘disgusting’. Thus, when given the autonomy to make food decisions, the majority reported choosing less healthy foods and tended to exclude fruits and vegetables from their meals. Additionally, some children reported that they would consume fruits and vegetables only when they were prepared in ways that they enjoyed eating. For example, they would consume vegetables only when the vegetables were prepared in ways that masked their taste or appearances, such as vegetables that are stir-fried with chili or topped with gravy. Nevertheless, a minority reported avoiding less healthy foods as they disliked the taste of less healthy foods (e.g. ‘too sweet, too salty’ and ‘too oily’) and not because of the benefits of healthy foods.

In a few FGD, some children have showed that they were aware of the nutritional value of foods but tended to rationalise their unhealthy eating habits in various ways. These children mentioned that consuming less healthy foods was acceptable if they had done, or would be doing, some physical activity on the same day. A few claimed that eating fries were considered healthy as fries were made from potatoes, a type of root vegetable. Additionally, a few children reported that their consumption of fruits and/or vegetables tended to ‘depend on (their) mood’ or their level of satiation. For example, they would not have fruits if they felt full after their main meal.

### Interpersonal influences

#### Parents’ influences on children’s accessibility to food and children’s attitudes and values towards food

Most children described their parents as gatekeepers to their food accessibility within, and sometimes out of, their homes. Although parents would buy unhealthy snacks when shopping for groceries, many children reported that their parents would restrict them from consuming these snacks. Some children also mentioned that their parents limit the frequency of their fast-food consumption as well. In response to these restrictions, a few children stated that they would adhere to them to appease their parents, while most found ways around these restrictions by consuming the restricted foods in secrecy. For example, purchasing snacks from convenient stores in secret and taking snacks from parent’s ‘secret stash’ of snacks without them knowing. Besides restricting less healthy foods, parents’ provision of healthy foods was also a common practice described by the children. This included preparing fruits and vegetables at home in ways that children prefer and using healthier cooking methods when preparing home meals. When eating out or buying take-outs, some parents would also ensure that vegetables were included as part of the meal.

The children’s attitudes towards food were also reported to be influenced by their parents in various ways. Some recounted that their parents would impart health knowledge to get them interested in or be more aware of the benefits of healthy eating. One child narrated that his father taught him that too much unhealthy foods will cause arteries to be ‘clogged’ and can lead to death. Others mentioned that their parents would use coercive methods, such as reprimanding or scaring them, to get them to adhere to a healthy diet. For example, telling them that worms will ‘grow’ in them if they eat too many sweets. Some of the children also expressed displeasure at their parents’ constant nudging to consume fruits and vegetables, yet not doing so themselves. The children also tended to model after their parents’ dietary and snacking behaviours, whether healthy or unhealthy. Some expressed that snacking on unhealthy snack foods together with their parents was an enjoyable experience.

#### Peer influence

The influence from peers was brought up in some FGD. The responses from these children suggest that some of their food choices were in part influenced by their peers, whether positively or negatively, depending on the food selection and preferences of their companions. Some children revealed that they get access to snacks from their friends at times and they would accompany their friends to patronise fast food joints or convenience stores. Conversely, some children reported that peers also encouraged them to eat healthily by persuasion, for instance, ‘nagging’ or ‘forcing’ them to finish the vegetables on their plate during recess time and assuring them that the vegetables served are palatable. Most children also appeared to perceive that fast food consumption was a norm among children, and this made them less hesitant to consuming fast foods.

#### Teacher’s influence during meal and snack times

Teachers were reported to have influence over the children’s food choices and behaviours through education and monitoring. Besides teaching them about healthy eating as part of the school curriculum, some children also spoke of how their teachers would monitor their fruit and vegetable intakes during recess time and the types of snacks consumed during snack time. One child also mentioned that she had changed her food choice from *roti prata* (a type of fried bread) to fish ball noodles for recess after heeding her teacher’s recommendations. However, they revealed that such efforts were not consistent among teachers, as some were less strict in enforcing healthy eating habits (e.g. giving children warnings but still allowing them to consume less healthy snacks in class).

### Environmental influences

#### Health education in schools and school policies

As part of the school curriculum, children learned about healthy foods and their recommended portion sizes using My Healthy Plate^(27)^, during physical education lessons. Besides learning about healthy eating, schools also ensured a healthy food environment through the Healthy Meals in Schools Programme which provides guidelines on the provision of healthier food options in schools^(28)^. The children who had their meals in school were aware that fruits and vegetables were provided in all the meals sold in school. Furthermore, the children from one school mentioned that they were only allowed to bring ‘healthy’ snacks, such as fruits and wholemeal biscuits, for snack time in school. Despite the schools’ provision of healthier meals, not all children consumed the fruits and vegetables provided. Some children revealed that they would either give away the fruits and vegetables to their friends or throw them away if they were not to their liking.

#### Incentives and environmental cues in school

The children from one school reported that the inter-class fruits and vegetable consumption competitions organised by the school incentivised them to consume more fruits and vegetables in school. It also seemed that children from this school were more inclined towards consuming fruits and vegetables as compared with the school where such incentives were not present. However, a few children expressed that such initiatives were not sustainable, as they noticed that their peers would stop consuming fruits and vegetables when the competition ended. The children from both schools also noted the presence of posters in their schools that reminded them about healthy eating. However, despite having these posters at areas frequented by the children (e.g. the canteen), the children from one focus group observed that not many of their peers paid attention to the messages on the posters.

#### Food accessibility in the neighbourhood

The children also highlighted how their neighbourhood and environment around the school influenced the access that they had to various foods. For example, the proximity of fast-food chains and convenience stores to their schools, homes or even the routes that they took to get home from school allowed them easy and quick access to less healthy foods or snacks. Children from another focus group also revealed that they would purchase snacks from a convenience store ‘just across the street’ from their school.

### Macrosystem influences

#### Health promotion advertisements

In a few focus groups, there was mention of posters containing health messages (e.g. posters from the Let’s Beat Diabetes campaign^(29)^) that could be found ‘everywhere in Singapore’, which reminded the children to eat healthily. Some of them mentioned picking up information about their health through advertisements on digital platforms, such as YouTube, which acted as an alternative source of health knowledge to the children outside of school. Particularly, a few children from one FGD demonstrated the lasting impression of one of the video advertisements from the Let’s Beat Diabetes campaign by re-enacting it.

## Discussion

Our findings through children’s perspectives showed that the influences on their dietary behaviours were from multiple levels of the sem, separately as well as interacting across levels.

While the children in our study appeared to know the ‘hows and whys’ of healthy eating, this knowledge alone did not necessarily translate into consistent healthy dietary behaviour. The importance of health and nutrition appeared to be a low priority among the children in our study. Instead, taste and appearance preferences appeared to be pivotal influences over their food decisions. These findings match those observed in previous studies conducted with children and adolescents in other populations which showed that the taste, smell, texture, appearance, as well as their emotional attachment to foods (e.g. pleasure and disgust) were more influential on their food choices than the nutritional value of food^(10,11)^. It has been suggested that at this age, the concept of nutrition and diet-related diseases may be too abstract to comprehend or perceived as distant and irrelevant^(30)^. Furthermore, enjoying the immediate gratification from consuming less healthy foods may often outweigh waiting out the long-term benefits of healthier eating^(31)^. This reinforces the need to focus on the short-term benefits and effects of healthy and unhealthy eating, respectively, when messaging health promotion interventions to children, rather than the longer term health implications. To motivate healthier dietary behaviours among children, some studies that changed children’s hedonic response towards fruits and vegetables (e.g. making healthy foods fun and enjoyable or used incentives, gamified healthy eating and experiential learning) have shown promising outcomes^(32–34)^. Similar effects have also been reported among recent Asian studies, suggesting that such activities could be adapted to interventions targeting children in Singapore^(35–37)^.

Despite the awareness of the low nutritional value of less healthy food, children in our study reported justifying their unhealthy eating habits based on their beliefs of certain foods (e.g. categorising fries as a vegetable) and actions (e.g. compensating unhealthy food consumption with physical activity). A possible explanation could be due to the cognitive dissonance between their knowledge and food preferences, thus resulting in the formation of beliefs to minimise this conflict and feelings of guilt when consuming less healthy foods^(38)^. Although some of these beliefs could motivate one to engage in healthy behaviours (e.g. physical activity), evidence has shown that they were often associated with poorer health outcomes among adults and adolescents^(39)^. Currently, there is limited research exploring this among children, further research is required to better understand children’s cognitive process and biases towards healthy and unhealthy foods and their health beliefs. These behaviours could also be due to the lack of procedural nutrition knowledge (i.e. knowing how to practice healthy eating) among children as the current knowledge students reported tended to be more declarative (i.e. knowing what healthy eating is). This highlights the importance of enhancing the procedural and declarative nutrition knowledge of children^(40)^.

On the interpersonal level, children in our study generally viewed parents as being quite influential in their dietary choices and frequently cited parents as the gatekeepers to the food supply at home. A range of food parenting practices have been reported by children in our study. Some of these practices have shown to help encourage healthy eating among children, while others appear to be counterproductive, such as setting restrictions on unhealthy food consumption which resulted in them eating in secrecy, as reported by children in our study. This finding is consistent with those in earlier studies which showed that the restriction of these ‘forbidden foods’ tended to increase children’s desire to consume these foods in the short-term and may contribute to dysregulated eating behaviours in the long term^(41)^. While it is important to limit unhealthy food consumption in children, teaching children to self-regulate their food consumption by providing guidance and routines, setting limits and considering the child’s perspectives may be better alternatives^(41)^. Our findings also highlighted the importance of parents’ role modelling on fruit and vegetable consumption as contradictory behaviours of parents could undermine children’s perceived importance of adherence to healthy eating^(42)^. This suggests that besides enhancing parents’ skills to feed their children healthily, parents should be encouraged to recognise how influential their eating behaviour is and practice positive role modelling. Given the strong influence of parental practices on children’s dietary behaviour, and the scarcity of such research among Asian parents^(43–45)^, further research is warranted to understand the food parenting practices and children’s responses to them among the Asian population.

Besides parental influences, influences from school have shown to have a significant impact on children’s food choices and dietary behaviour, such as encouraging and enforcing the consumption of healthy foods like fruits and vegetables in school. Existing research has observed that children whose schools provided healthy foods and drinks tended to consume more healthy foods and have lower BMI^(15)^. Hence, such policies to ensure the availability of healthy foods and drinks in schools should be continued. However, similar to a previous survey study, our findings also suggested that the provision of healthier food options in school does not necessarily increase the intake of these foods in all children as some children would still find ways to avoid eating them^(46)^. The consumption of healthier foods, such as fruits and vegetables, seemed to be primarily driven by children’s personal preferences or affect towards the food, as shown in our findings and previous study among Indian children^(47)^. As previously mentioned, the provision of incentives, gamifying healthy eating and experiential learning could be alternative ways to garner children’s interest^(32–34)^. Providing more variety of healthy foods that are prepared in a hygienic way could also help increase children’s affect towards healthy foods^(47)^. Apart from communication via school programmes, it is also crucial to ensure consistency in reinforcing healthy eating among teachers and across home and school as conflicting messages may confuse children and hinder their ability to make prudent dietary choices.

Peer influence has been found in previous literature to have a strong influence on children’s food acceptability and selection as children desire to seek approval from their peers and conform to the group^(7,14)^. However, the influence from peers was not frequently mentioned in our study, which is an unexpected finding. A possible explanation could be due to the limited time children spend with their friends over school meals. Unlike their Western counterparts whose school curriculum includes a 1-h lunch break, children in Singapore typically have a 30-min recess break, during which some children would spend their time playing instead of having a meal. Previous studies have also found that parents appear to be more influential to children between the ages of 10 and 14, and they may be less concerned about what their peers think^(48)^. These findings suggest that more focus should be put on parents than peers in interventions among children of this age group in Singapore.

Our findings also highlighted some important influences from the environmental and macrosystem levels of the sem, such as the proximity of stores and restaurants that sell unhealthy foods and the presence of health advertisements around them. Although the presence of these stores may not directly lead children to eat less healthy foods, their easy access coupled with children’s heightened preference for these foods may encourage the consumption of such foods^(16)^. Past research suggests that modifying the built environment, such as the implementation of zoning laws to limit the number or proximity of fast-food chains and convenience stores in residential areas or near schools, could help reduce the consumption of less healthy foods among children^(16)^. However, such interventions may be difficult to achieve in Singapore as shops, schools and housing are closely nested together due to the small island size. More research is needed to explore how such health concerns and interventions can be aligned with urban planning policies in Singapore. Finally, contrary to expectations, the influence of media on encouraging less healthy food consumption was not mentioned among our participants. Instead, the use of media to encourage healthy eating was reported. This could be attributed to the effect of regulations on food advertising to children in Singapore^(49)^, as well as the increase in health promotion advertisements in Singapore after the local Ministry of Health declared ‘War on Diabetes’ (a nationwide campaign aimed at lowering diabetes incidence rates) in 2016^(29)^. Although our findings suggest that children did not pay heed to the messages of the health promotion posters in school, the advertisements on the online platform seemed to capture their attention. Given the high media consumption and social media usage among children in Singapore^(50)^, future health promotion messages to target primary school children could be delivered on online platforms frequented by children.

To the best of our knowledge, this is the first study to examine children’s perspectives of the influences on their dietary behaviour in Singapore. The use of qualitative methods with children directly allowed us to gather rich data from them through their perspectives, therefore contributing to the limited but growing body of literature in Asia^(10,11)^ and locally^(43,44)^. However, there are some limitations to be considered. One limitation of the present study is that most of the children who participated were of Chinese descent, thus their experiences may not be generalised to all primary school children in Singapore, which consists of a racial mix of Chinese, Malay and Indian children. Besides, information on the socio-economic status of the children’s families was not collected. Thus, when conducting our analysis, we were unable to elicit information on the influence of socio-economic status on the children’s dietary behaviour. Further research is warranted to explore the views of children of Indian and Malay descent, as well as the influence of socio-economic status on the influences. It should also be noted that in 3 of the focus groups there were only 2 participants instead of the recommended minimum of 4 participants per group. Despite having fewer participants, we noticed that the children had the opportunity to provide more depth and detail through their interaction with each other. As mentioned in our findings, younger children (9–10 years old) often gave more literal information and did not provide further elaboration. In such cases, the moderator had to probe using more direct questioning, which could unintentionally have resulted in the use of leading questions. Future research working with younger children could consider using more participatory-based methods, such as photovoice, drawing or storytelling^(51)^, to elicit more in-depth responses from children of this age group.

## Conclusion

Understanding the determinants of a healthy diet contributes to the foundation of effective interventions. The insights from children in this study can be used to inform the development of future lifestyle interventions and policies targeting the different levels of sem to promote healthy eating in children aged 9–12 years old. Our findings also demonstrated the value of eliciting children’s input to identify intervention gaps, suggesting that the development of interventions ‘made by children for children’ may be more well accepted by children. Although gathering data from children themselves would help in the development of a more relevant intervention, it is essential to consider the viewpoints of parents and schools, who are also important agents of socialisation in influencing children’s behaviour.

## Supplementary material

For supplementary material accompanying this paper visit https://doi.org/10.1017/S1368980022000404. click here to view supplementary material
