# Supplementary material for: Understanding children’s perspectives of the influences on their dietary behaviours
Source: Public Health Nutr. 2022 Feb 21;25(8):2156–66. doi: 10.1017/S1368980022000404 (PMC9991721; doi:10.1017/S1368980022000404)
Supplement: Supplementary file 1 [file S1368980022000404sup.zip › S1368980022000404sup001.docx]

Supplementary Figure. Participant flow of the study
