# Supplementary material for: Understanding children’s perspectives of the influences on their dietary behaviours
Source: Public Health Nutr. 2022 Feb 21;25(8):2156–66. doi: 10.1017/S1368980022000404 (PMC9991721; doi:10.1017/S1368980022000404)
Supplement: Supplementary file 1 [file S1368980022000404sup.zip › S1368980022000404sup002.docx]

**Supplementary Table: Key questions asked during focus group discussions**

| **SEM level** | **Key Questions** |
| --- | --- |
| Individual | - *(Choose from a pile of food cards)* What do you usually eat for lunch and dinner? - *(Choose from a pile of food cards)* Which are the food that you like to eat? Why do you like them? - What do you think about healthy eating? Do you think it is important? - How did you learn about healthy eating? |
| Social environment | - Who do you usually eat your meals/ snacks with? - Who decides where and what you eat usually? - *(Choose from a pile of people cards)* Who are the people who would normally encourage/ discourage you to eat more fruits and vegetables? Why do you think they would say this? - Do you have any rules at home or school regarding consuming snacks? |
| Physical environment | - How do you find the foods in your school canteen? - How do you usually get your snacks? |
